# Novel Mutations in *GPR68* and *SLC24A4* Cause Hypomaturation Amelogenesis Imperfecta

**DOI:** 10.3390/jpm12010013

**Published:** 2021-12-28

**Authors:** Figen Seymen, Hong Zhang, Yelda Kasimoglu, Mine Koruyucu, James P. Simmer, Jan C.-C. Hu, Jung-Wook Kim

**Affiliations:** 1 Department of Pedodontics, Faculty of Dentistry, Istanbul University, Istanbul 34116, Turkey; fseymen@istanbul.edu.tr (F.S.); yeldakasimoglu@gmail.com (Y.K.); mine.yildirim@istanbul.edu.tr (M.K.); 2 Department of Biologic and Materials Sciences & Prosthodontics, School of Dentistry, University of Michigan, Ann Arbor, MI 48109, USA; zhanghon@umich.edu (H.Z.); jsimmer@umich.edu (J.P.S.); janhu@umich.edu (J.C.-C.H.); 3 Department of Pediatric Dentistry, School of Dentistry & DRI, Seoul National University, Seoul 03080, Korea; 4 Department of Molecular Genetics, School of Dentistry & DRI, Seoul National University, Seoul 03080, Korea

**Keywords:** whole exome sequencing, pH sensing, ion transportation, hereditary enamel defects, hypomaturation

## Abstract

Amelogenesis imperfecta (AI) is a rare genetic condition affecting the quantity and/or quality of tooth enamel. Hypomaturation AI is characterized by brownish-yellow discoloration with increased opacity and poorly mineralized enamel prone to fracture and attrition. We recruited three families affected by hypomaturation AI and performed whole exome sequencing with selected individuals in each family. Bioinformatic analysis and Sanger sequencing identified and confirmed mutations and segregation in the families. Family 1 had a novel homozygous frameshift mutation in *GPR68* gene (NM_003485.3:c.78_83delinsC, p.(Val27Cysfs*146)). Family 2 had a novel homozygous nonsense mutation in *SLC24A4* gene (NM_153646.4:c.613C>T, NP_705932.2:p.(Arg205*)). Family 3 also had a homozygous missense mutation in *SLC24A4* gene which was reported previously (c.437C>T, p.(Ala146Val)). This report not only expands the mutational spectrum of the AI-causing genes but also improves our understanding of normal and pathologic amelogenesis.

## 1. Introduction

Tooth enamel is the hardest tissue in the human body, covering the crown of the tooth not only to protect the underlying dentin–pulp complex but also to enhance esthetics and facilitate masticatory function. The formation of enamel begins with the ameloblasts’ secretion of enamel matrix proteins on the predentin, which triggers the growth and elongation of enamel crystallites [1]. The process continues until the full thickness of the enamel is achieved although only 30% of mineral by weight is produced. When the ameloblasts enter the maturation stage, they cycle through the ruffle-ended and smooth-ended phases during which matrix proteins are further degraded and removed and rapid appositional growth of enamel is accelerated [2]. The coordinated pH sensing and ion transportation of ameloblasts are critical for optimal enamel maturation [3,4].

Amelogenesis imperfecta (AI) is a heterogeneous collection of hereditary defects affecting tooth enamel. Depending on how the hereditary defect impacts the specific stage of enamel formation, AI is classified into hypoplastic, hypocalcified, and hypomatured types [5]. AI-affected individuals suffer from poor esthetics and masticatory discomfort including hypersensitivity to cold and hot stimuli and marked impact on the psychosocial wellbeing, such as social avoidance, distress, and low self-esteem [6,7].

Since the discovery of the amelogenins in the enamel matrix [8,9], many critical players in amelogenesis have been identified by biochemical analyses and genetic studies. To date, more than 20 genes are known to cause AI. Briefly, *AMELX* (amelogenin, OMIM *300391) [10], *ENAM* (enamelin, OMIM *606585) [11], *AMBN* (ameloblastin, OMIM *601259) [12], *RELT* (RELT TNF receptor, OMIM *611211) [13], and *ACP4* (acid phosphatase 4, also known as acid phosphatase, testicular; OMIM *606362) [14] are related to hypoplastic AI; *FAM83H* (family with sequence similarity 83 member H, OMIM *611927) is involved in hypocalcified AI [15]; and *ODAPH* (odontogenesis associated phosphoprotein, also known as *C4orf26*; OMIM *614829) [16], *MMP20* (matrix metalloproteinase 20, OMIM *604629) [17], *KLK4* (kallikrein related peptidase 4, OMIM *603767) [18], *WDR72* (WD repeat domain 72, OMIM *613214) [19], *SLC24A4* (solute carrier family 24 member 4, OMIM *609840) [20], and *GPR68* (G protein-coupled receptor 68, OMIM *601404) [21] are involved in hypomaturation AI.

In this study, we recruited three Turkish families with hypomaturation AI and performed mutational analyses with whole exome sequencing. The analyses revealed a novel frameshift mutation caused by an indel (insertion and deletion) in the *GPR68* gene and a novel nonsense mutation and a previously reported missense mutation in the *SLC24A4* gene. This report confirms the functional importance of GPR68 and *SLC24A4* in maturation amelogenesis, contributes various enamel phenotypes, and expands the mutational spectrum to advance our understanding of enamel maturation.

## 2. Materials and Methods

### 2.1. Study Subject Enrollment

The study protocol was independently reviewed and approved by the institutional review boards of the Seoul National University Dental Hospital (CRI05003G and 10 December 2020), Istanbul University (No: 2008/931 and 20 September 2019), and the University of Michigan (H03-00001835-M1 and 6 May 2021). Informed consent was obtained from the participating family members. Clinical examinations were performed, and saliva samples were collected.

### 2.2. DNA Isolation and Whole Exome Sequencing

Genomic DNA was isolated from the saliva samples, and the quality and quantity were measured. The DNA samples of selected individuals from each family (Table S1) were submitted to the Center for Inherited Disease Research at the Johns Hopkins University (CIDR, Baltimore, Maryland). The exomes were captured with the Agilent SureSelect Human All Exon Enrichment System. The sequencing reads were generated using the Illumina HiSeq 2500 (Illumina, Inc., San Diego, CA, USA).

### 2.3. Bioinformatics

The obtained 125-bp paired-end sequence reads were aligned to the reference human genome assembly (hg37) with the Burrows-Wheeler Aligner [22]. Sequence variants were obtained after the application of a series of bioinformatics analysis programs including Samtools and Genome Analysis Tool Kit [23,24]. Annovar was used to annotate the sequence variants with dbSNP build 147. A minor allele frequency (MAF) of 0.01 was the cutoff value for variant filtering.

### 2.4. Sanger Sequencing

The identified mutations in the probands and the segregation among family members were interrogated by Sanger sequencing using the following primers: *GPR68* exon 2 for family 1 (sense 5′-CCTTTCCTGCCTCTGACTTTC-3′ and antisense 5′-AGCACGTACTGCAGCCAGA-3′), *SLC24A4* exon 7 for family 2 (sense 5′-GTGGCCTGGAGTTAGGAGGT-3′ and antisense 5′-AGTGCCAGGGGCAGAGAT-3′), and *SLC24A4* exon 5 for family 3 (sense 5′-TTGGCTGTAGAGCGTCCAGT-3′ and antisense 5′-TGAGGCTCAGAGCTGACAAA-3′). Sanger sequencing was performed for all participating family members at Eurofins Genomics (Louisville, KY, USA).

## 3. Results

### 3.1. Family 1

**Figure 1:**
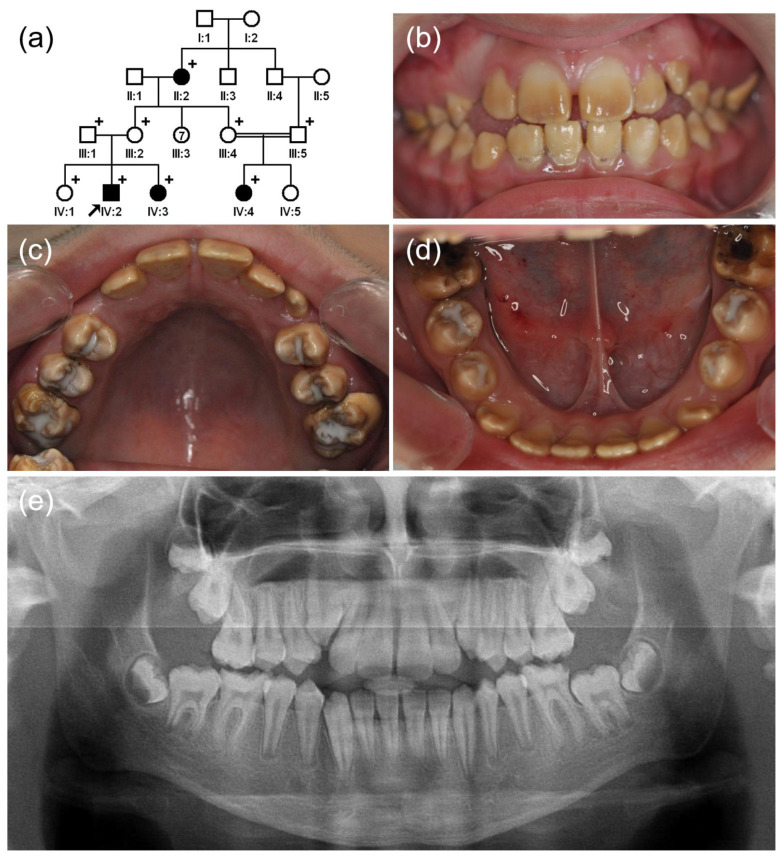
(**a**) Pedigree of family 1. The black arrow denotes the proband. Plus sign indicates participating individuals in this study. Number in the symbol indicates the number of siblings. Consanguineous marriage is shown by a double line. (**b**–**d**) Clinical photos of the proband (IV:2). Hypomatured enamel exhibits a brown discoloration, and the posterior teeth are treated with glass ionomer. (**e**) Panoramic radiograph shows hypomineralized enamel with a reduced radiodensity.

Family 1 was a four-generation Turkish family with consanguineous marriage (Figure 1). There were several affected individuals in generation II and IV, suggesting an autosomal recessive inheritance pattern. The proband was an 11-year-old second child (IV:2) from a non-consanguineous marriage, and he had no remarkable past medical history, except for the generalized brown discoloration of his dentition. The discoloration was noticed in all teeth but was less severe in the cervical region of the anterior teeth. Panoramic radiograph did not show a clear contrast between the enamel and dentin, suggesting that the enamel was hypomineralized. His 5-year-old younger sister (VI:3) was also affected (Figure S1). The maternal grandmother (II:2) and a cousin (IV:4) from a consanguineous marriage were similarly affected.

Mutational analysis of the whole exome sequencing data revealed a novel homozygous mutation in the *GPR68* gene (Figure S2) which was previously reported to cause autosomal recessive hypomaturation AI without any other syndromic phenotype. The identified mutation was a deletion of six nucleotides (GGTCTA) at position 78-83 in the cDNA and an insertion of a nucleotide (C) (NM_003485.3:c.78_83delinsC), and this would shift the translational frame to result in a truncated protein (NP_003476.3:p.(Val27Cysfs*146)). This mutation was not listed in the Exome Aggregation Consortium (ExAC) database (https://exac.broadinstitute.org/ (accessed on 27 November 2021)) or Genome Aggregation Database (gnomAD: https://gnomad.broadinstitute.org/ (accessed on 27 November 2021)).

### 3.2. Family 2

**Figure 2:**
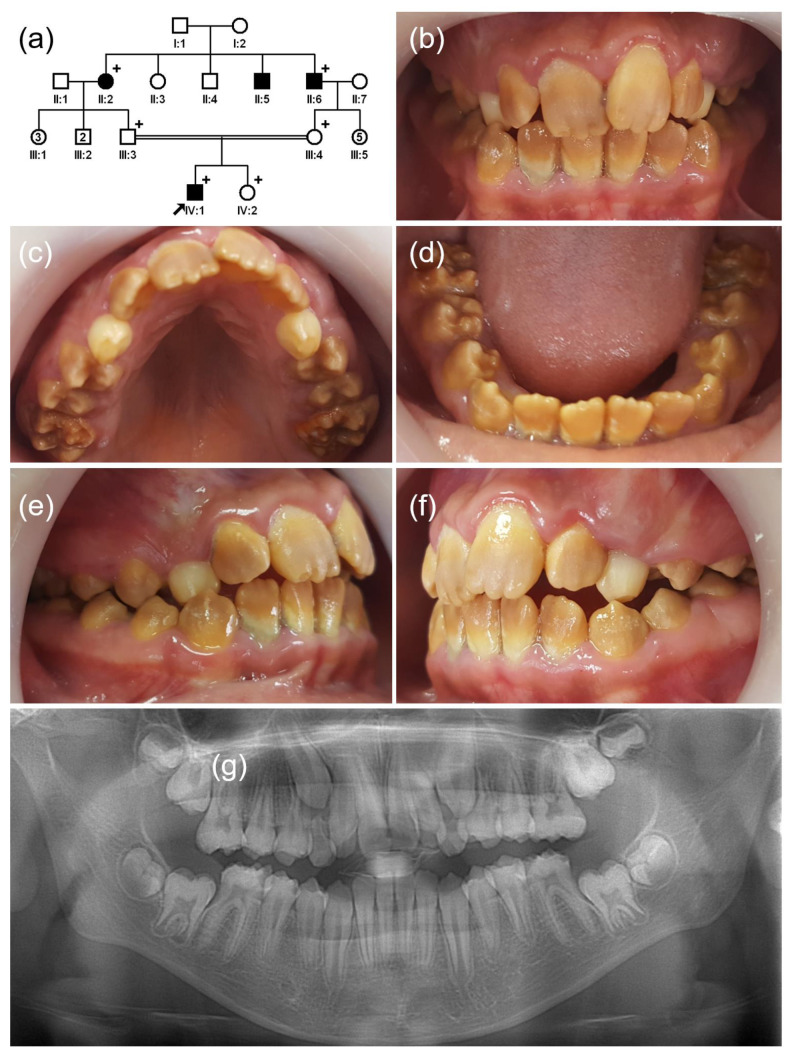
(**a**) Pedigree of family 2. The black arrow denotes the proband. Plus sign indicates participating individuals in this study. Number in the symbol indicates the number of siblings. Consanguineous marriage is shown by a double line. (**b**–**f**) Clinical photos of the proband (IV:1). Hypomatured enamel exhibits a generalized brown discoloration. (**g**) Panoramic radiograph shows hypomineralized enamel with reduced radiodensity. Breakdown of weak enamel can be seen in the first molars.

The proband of family 2 was a 10-year-old first child (IV:1) from a consanguineous marriage in a four-generation Turkish family with hypomaturation AI (Figure 2). His dentition had a generalized brown discoloration, and the weak enamel on the first permanent molars had broken off. His younger sister (IV:2) was not affected, and they had no remarkable past medical histories. There were additional affected family members in generation II (II:2, II:5, and II:6), suggesting an autosomal recessive inheritance pattern.

Mutational analysis of the whole exome sequencing data with an autosomal recessive inheritance model revealed a novel homozygous mutation in *SLC24A4* gene (Figure S3), which was previously proven to cause non-syndromic hypomaturation AI. The identified mutation was a nonsense mutation (NP_705932.2:p.(Arg205*)) caused by a transitional change of a cytosine to a thymine in exon 7 (NM_153646.4:c.613C>T). The mutation would have resulted in the decay of the mRNA by the nonsense-mediated surveillance system because the truncating mutation was in the early exon among the 17 coding exons [25,26]. The mutation was listed in the dbSNP database as rs141131742 and in gnomAD with an allele frequency of 2/251316.

### 3.3. Family 3

**Figure 3:**
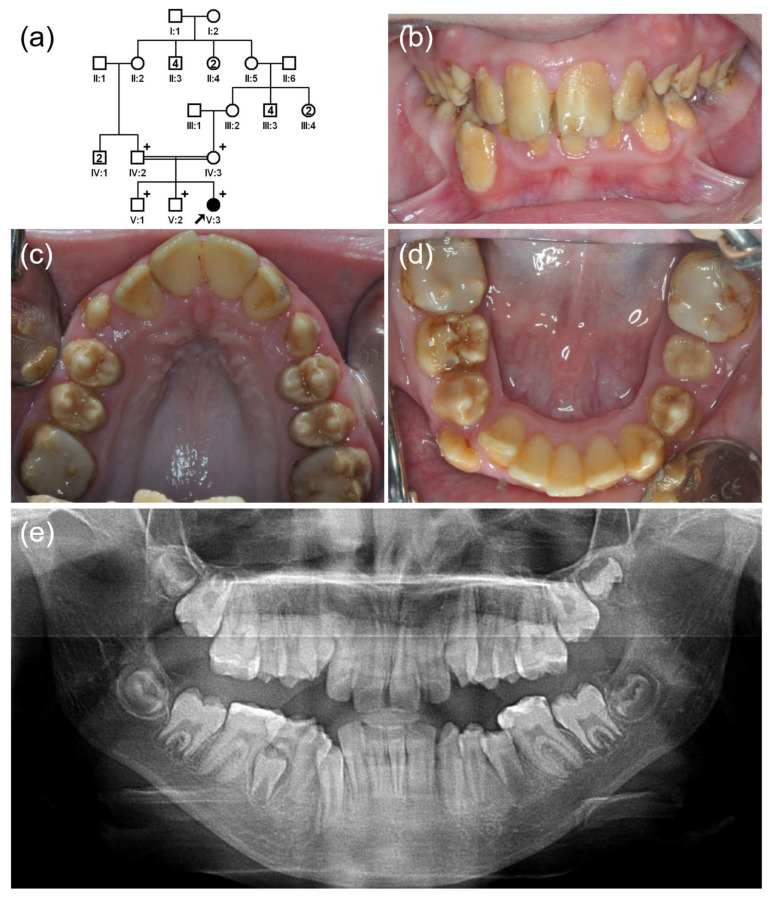
(**a**) Pedigree of family 3. The black arrow denotes the proband. Plus sign indicates participating individuals in this study. Number in the symbol indicates the number of siblings. Consanguineous marriage is shown by a double line. (**b**–**d**) Clinical photos of the proband (V:3). Hypomatured enamel exhibits a brown discoloration, and the first molars are treated with a composite resin. (**e**) Panoramic radiograph shows hypomineralized enamel with a reduced radiodensity.

The proband of family 3 was a 10-year-old third child (V:3) from a consanguineous marriage in an extended five-generation Turkish family (Figure 3). The pregnancy and delivery of this child was uneventful, and there was no remarkable past medical history. Her teeth were brown, and the first molars were restored with composite resin due to the enamel breakdown probably resulting from masticatory force imposed on the weak, hypomineralized enamel.

There were no other family members with similar enamel defect. Therefore, the mutational analysis was performed with the possibility of a spontaneous mutation and autosomal recessive inheritance model. A homozygous missense mutation (NP_705932.2:p.(Ala146Val)) in the *SLC24A4* gene was identified (NM_153646.4:c.437C>T) (Figure S4). This mutation was listed in the dbSNP database as rs587777537 and was previously reported to cause non-syndromic autosomal recessive hypomaturation AI.

## 4. Discussion

*GPR68* is one of the proton-sensing membrane receptors belonging to the G protein-coupled receptor (GPCR) superfamily [27]. There are more than 800 GPCRs in humans, and they are involved in diverse biological functions that sense and relay information from the extracellular to the intracellular environment [28]. Interestingly, they share similar structural features, having an extracellular N-terminus, intracellular C-terminus, and in-between seven transmembrane domains, with three extracellular and three intracellular loops [27]. *GPR68* also has the same structure which interestingly is encoded by a single coding exon. There are 11 transcripts reported in the National Center for Biotechnology Information (NCBI) database (https://www.ncbi.nlm.nih.gov/ (accessed on 28 November 2021)), all with different noncoding exon(s). Even though the tissue-specific expression of each transcript remains to be further studied, the expression of the *GPR68* protein in the ameloblast and underlying stellate reticulum was confirmed previously [21,29].

The mutation identified in this study was a delin frameshift mutation that occurred early in the transcript (NM_003485.3:c.78_83delinsC) (Table 1). This mutant transcript would introduce an early termination codon and produce a truncated protein with only 27 N-terminus plus 145 novel amino acids (p.(Val27Cysfs*146)) because it could escape from a nonsense-mediated mRNA decay system [25,26]. However, the mutant protein could not function like the wild-type protein (365 amino acids) due to the loss of most of its structural components.

The clinical phenotype of *GPR68* mutations presented in a previous study was slightly different from the typical hypomaturation AI; the brown discoloration was minimal in some affected siblings [21]. However, the affected enamel was apparently of full thickness but poorly mineralized and showed early loss of opaque enamel due to fracture. The clinical phenotype of the proband of family 1 in this study presented with typical brown discoloration, except the near-normal color in the cervical region of the permanent anterior teeth (especially in the maxillary central incisors). Other affected individuals exhibited typical clinical features of brown-discolored hypomaturation AI (Figure S1).

Families 2 and 3 had mutations in the *SLC24A4* gene, which encodes a potassium-dependent sodium/calcium exchanger. Mutations in this gene have been proven to cause hypomaturation AI by disrupting the function of removing and supplying essential ions for the maturation of the enamel crystallites [20]. Additionally, it has been suggested to be associated with blond/brown hair and blue/green eyes [30]. The missense mutation identified in this study was previously reported [31]. Additionally, there were several previously reported loss of function mutations (Table 2). *SLC24A4* is a transmembrane protein belonging to a sodium/potassium/calcium exchanger superfamily with a cytoplasmic N-terminus, 11 transmembrane domains, and an extracellular C-terminus [32]. The superfamily has highly conserved shared hydrophobic domains (α-1 and α-2 repeats), and the p.(Ala146Val) mutation is in the α-1 repeat [33].

The clinical phenotypes of families 2 and 3 were similar to typical hypomaturation AI. There was a generalized dark brown discoloration in all teeth, and the poorly mineralized, relatively weak enamel tended to be prematurely lost to attrition or masticatory stress, especially in the posterior teeth.

In conclusion, we recruited three hypomaturation AI families, and mutational analyses revealed a *GPR68* mutation in family 1 and *SLC24A4* mutations in families 2 and 3. This is the second paper reporting a *GPR68* mutation and suggests that the phenotype caused by the *GPR68* mutation can be variable depending on the type of mutation and/or genetic background. Further functional studies with more mutation cases are needed to characterize the factors influencing the various clinical phenotype and to advance our understanding of the normal and pathologic enamel formation.

## Figures and Tables

**Table 1:** Disease-causing mutations in *GPR68* gene.

Location	cDNA	Protein	Mode of Inheritance	References
Exon 2	c.83_84del	p.(Tyr28Cysfs*146)	AR homo	This report
Exon 2	c.221T>C	p.(Leu74Pro)	AR homo	Parry et al. (2016) [21]
Exon 2	c.386_835del	p.(Phe129_Asn278del)	AR homo	Parry et al. (2016) [21]
Exon 2	c.667_668delAA	p.(Lys223Glyfs*113)	AR homo	Parry et al. (2016) [21]

Sequences based on the reference sequence for mRNA (NM_003485.3) and protein (NP_003476.3), where the A of the ATG translation initiation codon is nucleotide 1.

**Table 2:** Disease-causing mutations in *SLC24A4* gene.

Location	cDNA	Protein	Mode of Inheritance	References
Exon 5	c.437C>T	p.(Ala146Val)	AR homo	Wang et al. (2014) [31] This report
Exon 7	c.613C>T	p.(Arg205*)	AR homo	This report
Exon 11	c.1015C>T	p.(Arg339*)	AR homo	Parry et al. (2013) [20]
Exon 12	c.1192C>T	p.(Gln398*)	AR homo	Khan et al. (2020) [34]
Exon 13	c.1307T>G	p.(Leu436Arg)	AR homo	Herzog et al. (2015) [35]
Intron 14	deletion of 10042 bp	(loss of exons 15–17)	AR homo	Seymen et al. (2014) [36]
Exon 14	c.1495A>T	p.(Ser499Cys)	AR homo	Parry et al. (2013) [20]
Exon 15	c.1604G>A	p.(Gly535Asp)	AR homo	Lepperdinger et al. (2020) [37]

Sequences based on the reference sequence for mRNA (NM_153646.4) and protein (NP_705932.2), where the A of the ATG translation initiation codon is nucleotide 1.

## Data Availability

The data presented in this study are openly available in ClinVar (http://www.ncbi.nlm.nih.gov/clinvar (accessed on 27 November 2021)), Submission ID: SUB10711085 and SUB10726703.

## Supplementary Materials

The following are available online at https://www.mdpi.com/article/10.3390/jpm12010013/s1, Table S1: Statistics for exome sequencing; Figure S1: Clinical photos and panoramic radiograph of individuals IV:3 and IV:4 of family 1; Figure S2: Sequencing chromatograms of the participating individuals of family 1; Figure S3: Sequencing chromatograms of the participating individuals of family 2. Figure S4: Sequencing chromatograms of the participating individuals of family 3.
