# Supplementary material for: Novel Mutations in GPR68 and SLC24A4 Cause Hypomaturation Amelogenesis Imperfecta
Source: J Pers Med. 2021 Dec 28;12(1):13. doi: 10.3390/jpm12010013 (PMC8781920; doi:10.3390/jpm12010013)
Supplement: Supplementary file 1 [file jpm-12-00013-s001.zip › jpm-1505834-supplementary.pdf]

Novel Mutations in *GPR68* and *SLC24A4* Cause Hypomaturation Amelogenesis Imperfecta

Figen Seymen [1,†], Hong Zhang [2,†], Yelda Kasimoglu [1], Mine Koruyucu [1], James P. Simmer [2], Jan C.-C. Hu [2] and Jung-Wook Kim [3,4,*]

[1]Department of Pedodontics, Faculty of Dentistry, Istanbul University, Istanbul 34116, Turkey
[2]Department of Biologic and Materials Sciences & Prosthodontics, School of Dentistry, University of Michigan, Ann Arbor, MI 48109, USA.
[3]Department of Pediatric Dentistry School of Dentistry & DRI, Seoul National University, Seoul 03080, Korea.
[4]Department of Molecular Genetics School of Dentistry & DRI, Seoul National University, Seoul 03080, Korea.

† These authors equally contributed to this work.
* Correspondence: pedoman@snu.ac.kr (J.-W.K.)

Table S1. Statistics for exome sequencing.

Sample		Total reads	Mapping rate (%)	Median target coverage	Coverage of target region (%)	Fraction of target covered with at least	
						20X	10X
Family 1	III:1	234,165,508	99.7	131	99.5	98.3	99.1
	III:2	132,545,626	99.6	81	99.4	96.4	98.6
	IV:1	135,837,010	98.9	79	99.4	96.1	98.4
	IV:2	141,952,404	99.6	89	99.4	96.7	98.6
	IV:4	168,717,012	99.1	102	99.3	97.4	98.6
Family 2	III:3	119,361,758	99.9	78	99.5	96.1	98.5
	III:4	126,587,438	99.8	81	99.4	96.1	98.3
	IV:1	124,664,816	99.8	79	99.5	96.0	98.5
	IV:2	123,014,990	99.7	75	99.4	95.9	98.4
Family 3	IV:2	224,880,866	99.5	122	99.5	98.2	99.1
	IV:3	112,776,376	99.3	69	99.4	95.3	98.3
	V:1	140,832,940	99.8	88	99.4	96.8	98.6
	V:3	156,310,202	99.7	96	99.3	97.2	98.6

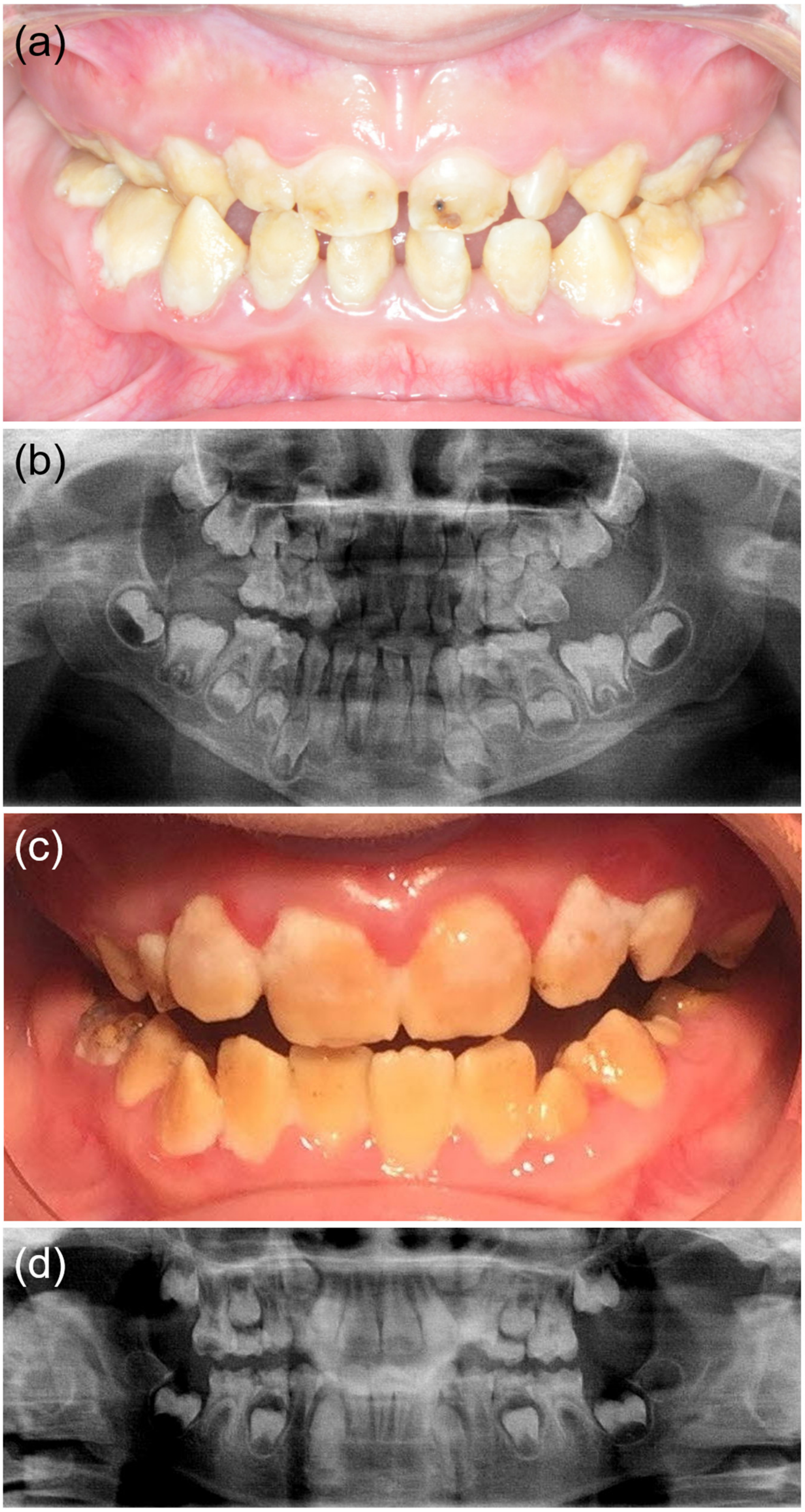

Figure S1. Clinical photo (**a**) and panoramic radiograph (**b**) of individual IV:3 of family 1 at age 5. Clinical photo (**c**) and panoramic radiograph (**d**) of individual IV:4 of family 1 at age 9.

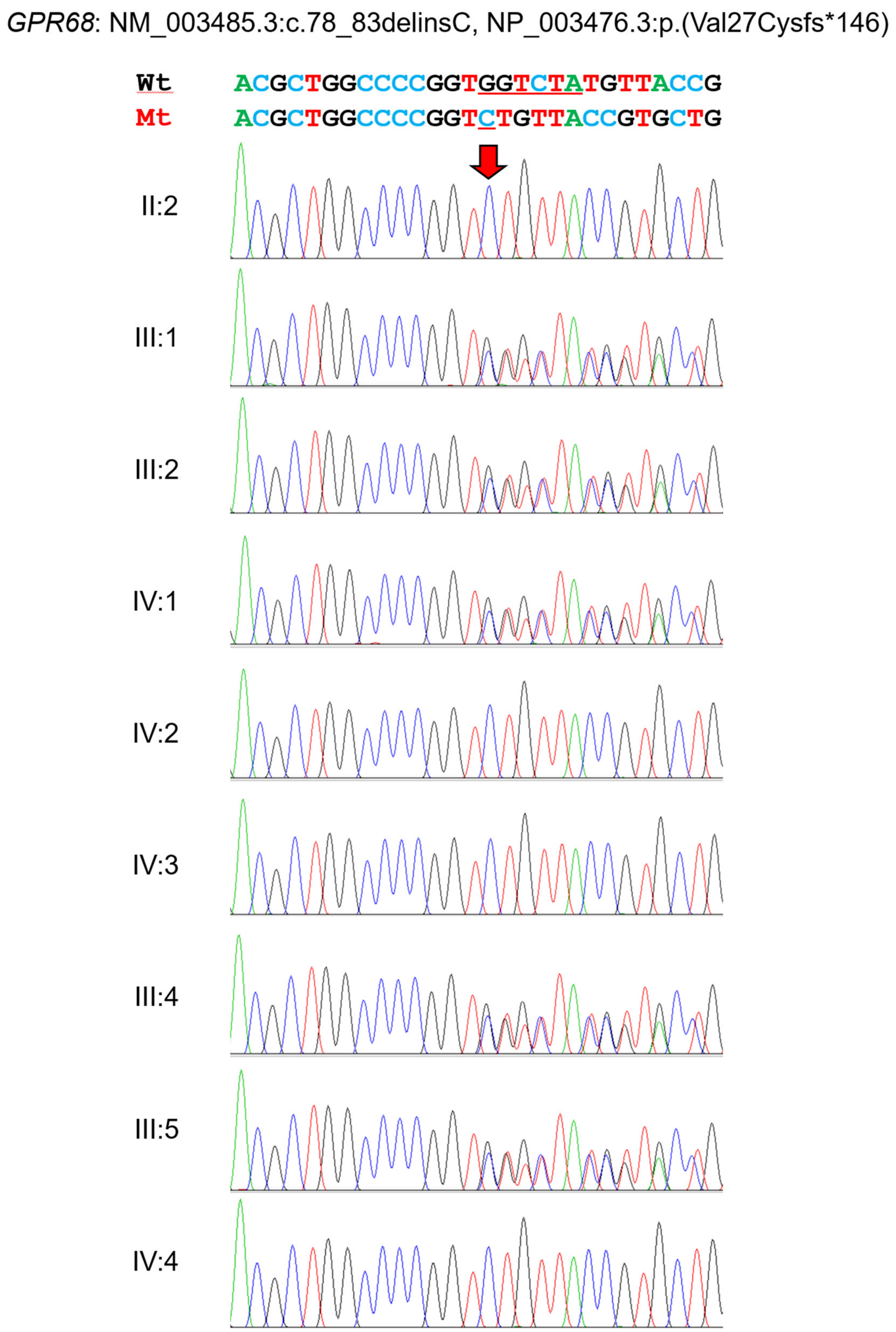

Figure S2. Sequencing chromatograms of the participating individuals of family 1. Wild type (Wt) and mutant (Mt) nucleotide sequences are shown above the chromatograms. Nucleotides affected by the mutation are underlined. The location of the mutation is indicated with a red arrow. Individual identifications are indicated on the left side of each chromatogram.

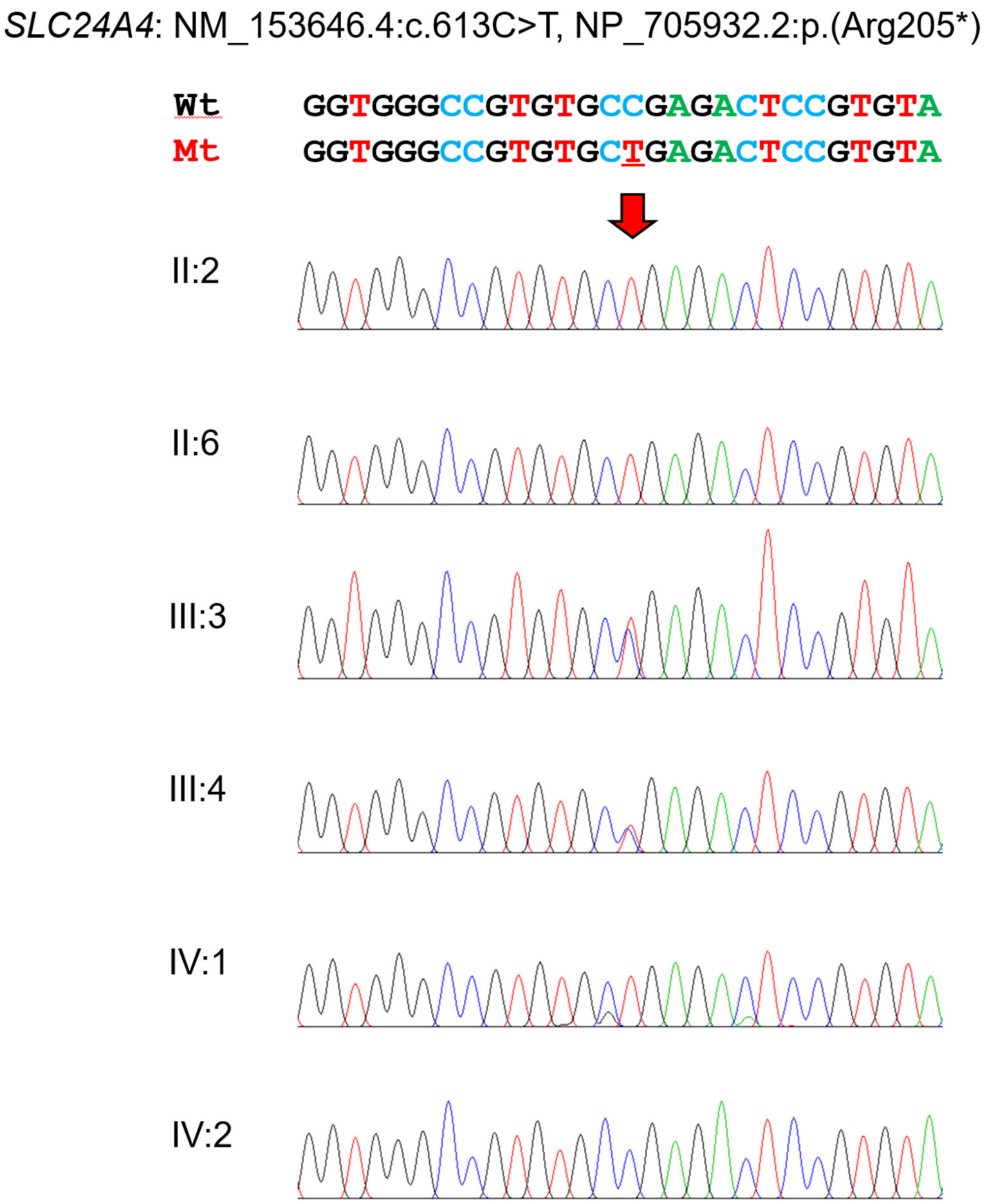

Figure S3. Sequencing chromatograms of the participating individuals of family 2. Wild type (Wt) and mutant (Mt) nucleotide sequences are shown above the chromatograms. Mutated nucleotide is underlined. The location of the mutation is indicated with a red arrow. Individual identifications are indicated on the left side of each chromatogram.

SLC24A4: NM_153646.4:c.437C>T, NP_705932.2:p.(Ala146Val)

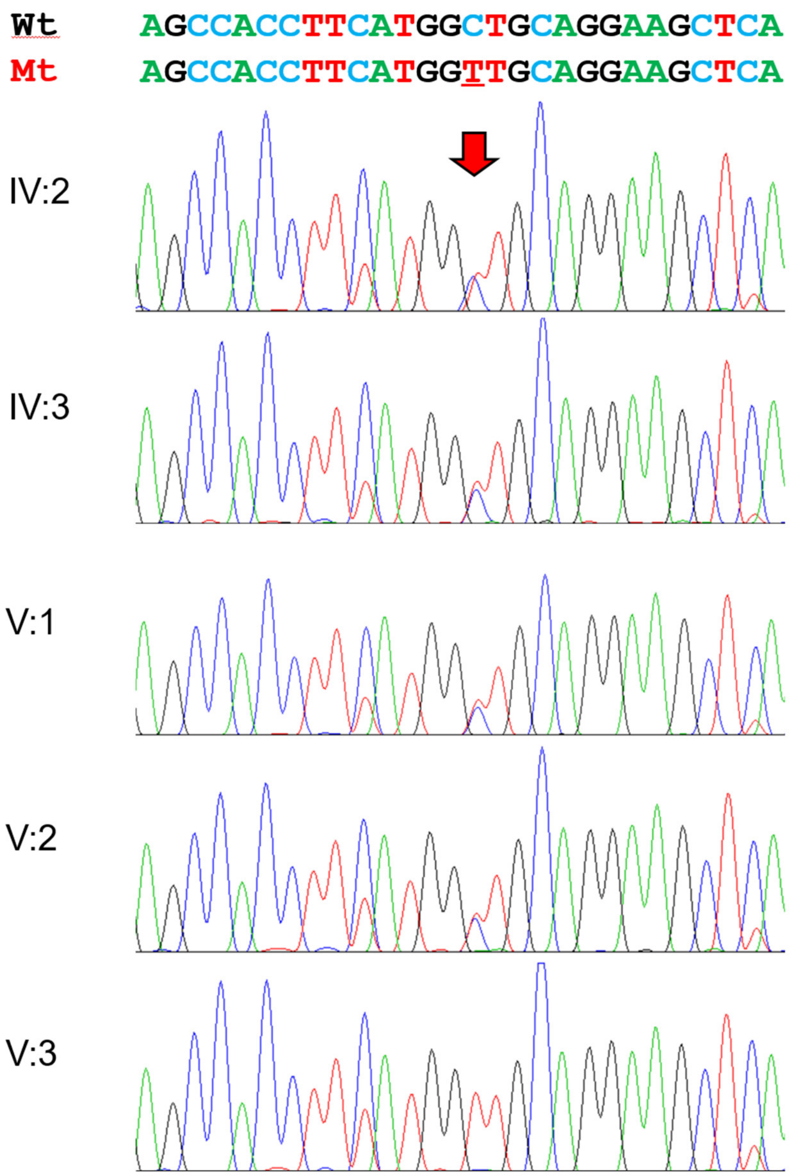

Figure S4. Sequencing chromatograms of the participating individuals of family 3. Wild type (Wt) and mutant (Mt) nucleotide sequences are shown above chromatograms. Mutated nucleotide is underlined. The location of the mutation is indicated with a red arrow. Individual identifications are indicated on the left side of each chromatogram.
